# MEMS Inertial Sensors Based Gait Analysis for Rehabilitation Assessment via Multi-Sensor Fusion

**DOI:** 10.3390/mi9090442

**Published:** 2018-09-03

**Authors:** Sen Qiu, Long Liu, Hongyu Zhao, Zhelong Wang, Yongmei Jiang

**Affiliations:** 1 School of Control Science and Engineering, Dalian University of Technology, Dalian 116024, China; liulong@neusoft.edu.cn (L.L.); zhaohy@dlut.edu.cn (H.Z.); wangzl@dlut.edu.cn (Z.W.); 2 Dalian Neusoft University of Information, Dalian 116023, China; 3 Dalian Medical University, Dalian 116027, China; natalie@mail.dlut.edu.cn

**Keywords:** MEMS sensors, gait analysis, rehabilitation assessment, body sensor network

## Abstract

Gait and posture are regular activities which are fully controlled by the sensorimotor cortex. In this study, fluctuations of joint angle and asymmetry of foot elevation in human walking stride records are analyzed to assess gait in healthy adults and patients affected with gait disorders. This paper aims to build a low-cost, intelligent and lightweight wearable gait analysis platform based on the emerging body sensor networks, which can be used for rehabilitation assessment of patients with gait impairments. A calibration method for accelerometer and magnetometer was proposed to deal with ubiquitous orthoronal error and magnetic disturbance. Proportional integral controller based complementary filter and error correction of gait parameters have been defined with a multi-sensor data fusion algorithm. The purpose of the current work is to investigate the effectiveness of obtained gait data in differentiating healthy subjects and patients with gait impairments. Preliminary clinical gait experiments results showed that the proposed system can be effective in auxiliary diagnosis and rehabilitation plan formulation compared to existing methods, which indicated that the proposed method has great potential as an auxiliary for medical rehabilitation assessment.

## 1. Introduction

Human walking contains important physiology, kinematic and dynamic information. There are many application prospects of human gait analysis in real life, such as monitoring the patient’s recovery progress in clinical practice, the control strategy of bionic robots, etc. For instance, stroke is a common neurodegenerative condition with a principal symptom of progressive limbs movement disorder. The prevalence of this neurological disease has been estimated at 120 affected individuals per 100,000 and 75% of the victims suffer from sequelae (mostly involve gait dysfunction) afterwards according to epidemiological statistics [1,2,3]. An insightful example of gait disorder can be observed in stroke—note that this study is not merely focused on stroke. However, a great majority of subjects who took part in the gait analysis experiment suffer from stroke. Furthermore, stroke patients share most gait symptoms with common neurological disorders in clinical practice. Specifically, stroke is a chronic neurological disorder associated with hemiplegia, lack of balance and abnormal gait. Therefore, objective and accurate inspection of gait parameters (as shown in Table 1) is of great help to a neurologist for appropriate assessment and diagnosis of stroke patients. To this end, the findings of this study could be helpful for revealing the pathogenesis of gait disorders. Stroke normally results in gait asymmetry, which can be reflected in the differences of gait phase partition between two feet due to paresis and imbalance. To sum up, it is crucial to discover the main components of gait disorders. In view of this situation, it is critical to develop an objective and quantitative approach to assess the patients’ physical condition. This paper established a wearable gait analysis platform based on a MEMSsensor and body sensor network. The platform can be used to collect acceleration, angular velocity and the geomagnetic signals in the process of walking movement. Accurate gait parameters can be calculated through a sensor data fusion algorithm and error correction process.

With the rapid development of modern medical technology, the concepts of medical treatment have been gradually changed to “Prevention first”. In this case, it is quite necessary to conduct the acquisition and processing of health information in advance, so that early medical diagnosis and intervention are feasible. Meanwhile, in order to implement ambulatory monitoring without affecting the subjects’ normal physiological activities, the traditional wire communication mode gradually shifts to a wireless mode, which is likely to be more persuasive. Moreover, innovative sensing technology is indispensable for continuous monitoring since the medical monitoring equipment has been developed towards microscale and a long-term span. To this end, the emerging Body Sensor Network (BSN) might serve as a peripheral node of the Internet of Medical Things or even the ubiquitous network.

## 2. Related Works

Quantitative gait analysis system mainly including a camera system [4,5,6], electromyography measuring system [7] and force platform [8,9]. The camera system consists of multiple high resolution cameras located in an indoor space, the orientation and position information of the target subject can be calculated using attached highlighting reflective spots. The electromyography measuring system detects human lower limb muscle signals by surface electromyography in the waking process; force platform reflects the change of plantar pressure during walking. However, the applications of above gait analysis system are limited in clinical practice, and the main reasons lie in three aspects. Firstly, the systems are expensive, which might be a barrier to routine use. Secondly, the usage of the systems are complex and require special operation, and it usually takes hours to complete the whole gait measurement process. Finally, specific space is normally needed to perform gait analysis using the above systems. In particular, the camera system may need more than 100 hundred square meters [10,11,12,13]. Table 2 lists a brief comparison of mainstream gait analysis methods.

Considerable research has been conducted into the progression of gait dysfunction through the various stages of stroke. Specifically, stroke subjects experience decreased stride length, cadence and walking speed, significant variability in stride length and gait cycle, and Walking imbalances [10,14,15,16]. Chang et al. [17] employed a specialized wearable system and found that stroke subjects demonstrated decreased gait velocity, stride length and prolonged double support phase. They further identified a high correlation between these gait parameters and age of onset. Further investigation into gait impairments in subjects with neurological diseases has also indicated that the degree of gait abnormality and the disease progression. Previous research has highlighted the advantages of quantitative gait analysis in gait diagnosis; however, laboratory-based systems such as optical tracking and plantar pressure measurement are typically expensive and are not available in ordinary clinical settings [18,19,20]. Therefore, significant interests have increased rapidly in the development of alternative gait analysis tools.

With the maturity of microelectromechanical systems and the development of information fusion technologies, the application of inertial motion analysis technology is becoming more and more extensive [8,21,22,23,24,25]. Due to the noticeable advantages of small size and low cost, wearable sensors can be mounted directly on the body segment with no need for specified test environment [24,26,27,28]. Such system may also serve as a good supplement of the gold standard including optical system and plantar pressure monitoring system. In previous studies, we have adopted a wearable inertial sensor in a walking distance calculation and walking pattern classification [29,30,31]. Ambulatory measurement of the participant’s trunk inclination using inertial measurement unit (IMU) was carried out by Farris et al. [3]. Bao et al. [32] developed a smart shoe for gait analysis using force sensitive resistors and IMU sensors. Luinge et al. [33] proposed the estimation of arm orientation by wearable inertial sensors. Dejnabadi et al. [34] introduced an approach to accurate measurement of joint angles based on IMU. However, due to their inability to detect heading reference, inertial based systems generally fail to measure differential orientation, a prerequisite for computing the 3D knee flexion angle recommended by the Internal Society of Biomechanics [35]. Roetenberg et al. [36] developed an ambulatory position and orientation tracking method fusing magnetic and inertial sensing. Since magnetometers measure the strength and direction of the local magnetic field, the geographical north direction can be found. In this case, the initial heading orientation can be obtained with the supplement of a magnetometer. Moreover, the system remains self-contained, which means it does not rely on any external infrastructure [37]. In addition, there are already wireless IMU BSN commercial products such as Trigno Avanti (Delsys Inc., Natick, MA, USA), Mvn Suit (XSens Inc., Enschede, The Netherlands), Perception Neuron (Noitom Inc., Beijing, China) and iSen (STT Systems Inc., San Sebastian, Spain). The current limitations of the state-of-the-art mentioned in the literature are the sensor alignment and integral error. Cost-effectiveness and stability are two other concerns. Moreover, little research about the follow-up monitoring of patients’ lower limbs has been carried out. Therefore, the contributions of this paper include the sensor alignment method and the availability of follow-up monitoring of patients’ key gait parameters.

The rest of this paper is organized as follows: Section 3 describes the structure of the proposed gait analysis system and the methodology used to estimate the gait parameters during walking; experimental results are given in Section 4; and the potential applications of gait analysis are discussed in Section 5, which concludes the paper as well.

## 3. Materials and Methods

### 3.1. System Setup

**Figure 1:**
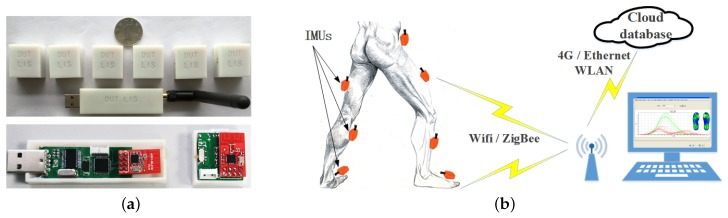
The principle and structure of the proposed gait analysis system (**a**) self-made motion tracking sensor nodes; (**b**) gait analysis scenario.

An ambulatory gait analysis system has been developed based on IMU. We have assembled the IMU from accelerometers/gyroscopes chipsets including ST Microelectronics (Geneva, Switzerland) and Analog Devices (Boston, MA, USA). A multi-sensor fusion algorithm is used to estimate gait parameters. The inertial measurement unit (IMU) can be strapped on both feet, shank and thigh via adjustable elastic straps with hook & loop. We chose straps over housing shells due to their flexible structure, strong adaptability, lower cost and good maneuverability. The installation is quite simple, which can be finished in several minutes. We have designed a fastener with which the sensor nodes can be fixed on the specified location firmly, ensuring the estimation accuracy. No special laboratory is needed, and the gait measurement can be performed just in the corridor or in the ward. The patients can even keep follow-up monitoring in the community after they discharged from the hospital. The performance-to-price ratio is relatively high and it is convenient to automatically generate a gait diagnostic report. Moreover, useful contrastive analysis can be made with repeated gait analysis. The principle and structure of the proposed gait analysis system is shown in Figure 1.

The sensor array includes triaxial accelerometers, gyroscope and magnetometer. Sensor performance specification is shown in Table 3. Raw sensor data is logged at 100 Hz and then forwarded to a receiver via a 2.4 GHz wireless network. The actual linear motion acceleration is used to calculate position. Note that the gravity component ($g={\left[0,0,9.81\right]}^{T}$m/s${}^{2}$) is normally eliminated from the resultant acceleration signal before estimating the position by integral operation of actual motion acceleration two times.

### 3.2. Accelerometer Non-Orthogonal Error Estimation

**Figure 2:**
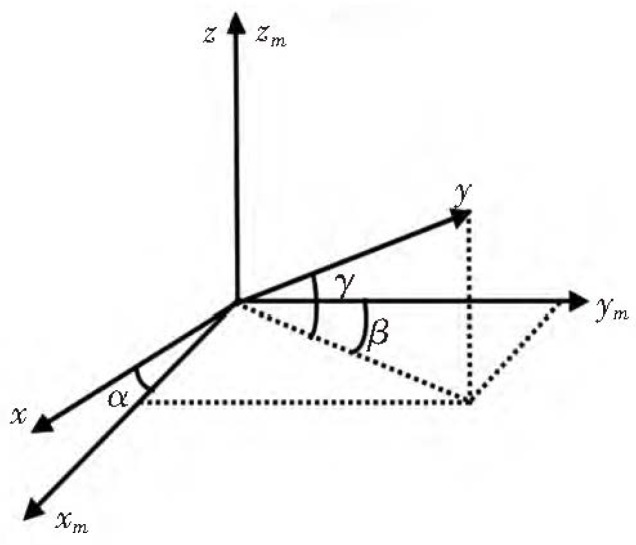
Non-orthogonal angle error of tri-axis accelerometer.

In general, the accelerometer orthogonal error is less than $1^{\circ }$, which is normally indicated in the product technical manual. Orthogonal errors exist in three-axis, as shown in Figure 2, and the non-orthogonal error can be expressed as follows:

(1) $$E=\left[\begin{array}{ccc} \cos\alpha & 0 & \sin\alpha \\ \sin\beta \cos\gamma & \cos\beta \cos\gamma & \sin\gamma \\ 0 & 0 & 1 \end{array}\right].$$

Formula (1) can be simplified using the approximation of the trigonometric function value and then we have the following equation:

(2) $$E\approx \left[\begin{array}{ccc} 1 & 0 & \alpha \\ \beta & 1 & \gamma \\ 0 & 0 & 1 \end{array}\right].$$

The accelerometer merely senses gravity under stationary state. A relationship exists between accelerometer observations $g_{s}$ and the true gravitational acceleration ±bg as follows:

(3) $$\;\pm \;bg=E^{-1}g_{s}=\left[\begin{array}{ccc} 1 & 0 & -\alpha \\ -\beta & 1 & \alpha \beta -\gamma \\ 0 & 0 & 1 \end{array}\right].$$

Meanwhile,

(4) $$\frac{g_{s}^{T}E^{T}Eg_{s}}{∥g_{s}{∥}^{2}}=1.$$

After establishing the non-orthogonal error correction model, the ellipsoid fitting method is introduced to calculate the non-orthogonal error angle. We can get three components of gravity vector ±bg with respect to three sensitive axes as follows:

(5) $$G_{g}=∥\;\pm \;bg∥{\left[-\sin\theta ,sin\phi \cos\theta ,\cos\phi \cos\theta \right]}^{T},$$

while the accelerometer observations $G_{s}=\left[a_{x},a_{y},a_{z}\right]T$ should satisfy the following equation theoretically:

(6) $$G_{s}^{T}G_{s}={∥\;\pm \;bg∥}^{2},$$

which means the distribution of measured values is a spherical with the radius of ${∥g∥}^{2}$; however, actually, the accelerometer measurement distribution is an ellipsoid due to the existence of nonorthogonal errors. Set $O={\left[a,b,c,f,g,h,p,q,r,d\right]}^{T}$ and quadric equation can be written as follows:

(7) $$ax^{2}+by^{2}+cz^{2}+2fyz+2gxz+2px+2qy+2rz+d=0.$$

Then, we can get the coefficient matrix from Equation (4) after ellipsoid fitting,

(8) $$\left[\begin{array}{ccc} a & d & c\\ d & b & f\\ e & f & c \end{array}\right]=\frac{E^{T}E}{∥g_{s}{∥}^{2}}.$$

Define A=a+b+c, $B=ab+bc+ac-f^{2}-g^{2}-h^{2}$, if $4B-A^{2}>0$. Then, Equation (7) is the description of ellipsoid surface. Select *n* group of accelerometer measurements, $\left[x_{i},y_{i},z_{i}\right]$, i=1,⋯,n, denote $C=[X_{1},X_{2},\cdots ,X_{n}]$ and $X_{i}={\left[x_{i}^{2},y_{i}^{2},z_{i}^{2},2y_{i}z_{i},2x_{i}z_{i},2y_{i}x_{i},2x_{i},2y_{i},2z_{i},1\right]}^{T}$, and then the ellipsoid fitting is converted into the following constraints:

(9) $$\left\{\begin{array}{c} 4B-A^{2}=1,\\ min(O^{T}C^{T}CO). \end{array}\right.$$

Define the coefficient matrix as follows:

(10) $$M_{0}=\left[\begin{array}{cccccc} -1 & 1 & 1 & 0 & 0 & 0\\ 1 & -1 & 1 & 0 & 0 & 0\\ 1 & 1 & -1 & 0 & 0 & 0\\ 0 & 0 & 0 & -4 & 0 & 0\\ 0 & 0 & 0 & 0 & -4 & 0\\ 0 & 0 & 0 & 0 & 0 & -4 \end{array}\right],$$

(11) $$M=\left[\begin{array}{cc} M_{0} & 0_{6\times 4}\\ \;\pm \;b0_{4\times 6} & \;\pm \;b0_{4\times 4} \end{array}\right].$$

Lagrangian function can be introduced to convert the constraint problem to the following equation:

(12) $$\left\{\begin{array}{c} O^{T}MO=1,\\ C^{T}CO=\lambda MO. \end{array}\right.$$

It has been proved that this constraint problem has a unique solution in the field of mathematics [38]. Therefore, the ellipsoid coefficient vector can be determined, and then the ellipsoid radius and the nonorthogonal error angle can be calculated based on the ellipsoid coefficient. Define the symmetrical coefficient matrix and the translation vector *S* $T={\left[2p,2q,2r\right]}^{T}$,

(13) $$S=\left[\begin{array}{ccc} a & h & g\\ h & b & f\\ g & f & c \end{array}\right].$$

Then, the transformation $H^{{}^{\prime }}=N^{T}H+R$ can convert the quadric equation into a standard ellipsoid equation:

(14) $$\frac{x^{2}}{a^{{}^{\prime }2}}+\frac{y^{2}}{b^{{}^{\prime }2}}+\frac{z^{2}}{c^{{}^{\prime }2}}=1,$$

where N is the eigenvector of matrix *S*, denote *D* as the main diagonal matrix of *S*, and then we have $S=NDN^{T}$ and $R=-{\left(2D\right)}^{-1}NT$. Ellipsoid radius can be obtained by the following formula:

(15) $$\left\{\begin{array}{c} a^{{}^{\prime }}=\sqrt{\frac{-{\left(NF\right)}^{T}R}{\left(2-d)D(1,1\right)}},\\ b^{{}^{\prime }}=\sqrt{\frac{-{\left(NF\right)}^{T}R}{\left(2-d)D(2,2\right)}},\\ c^{{}^{\prime }}=\sqrt{\frac{-{\left(NF\right)}^{T}R}{\left(2-d)D(3,3\right)}}. \end{array}\right.$$

After calculating the ellipsoid coefficient, we can calculate the nonorthogonal error angle by the analytic method. Equation (3) can be used to compensate the non-orthogonal error of the accelerometer.

### 3.3. Stance Phase Detection by Decision Level Data Fusion

**Figure 3:**
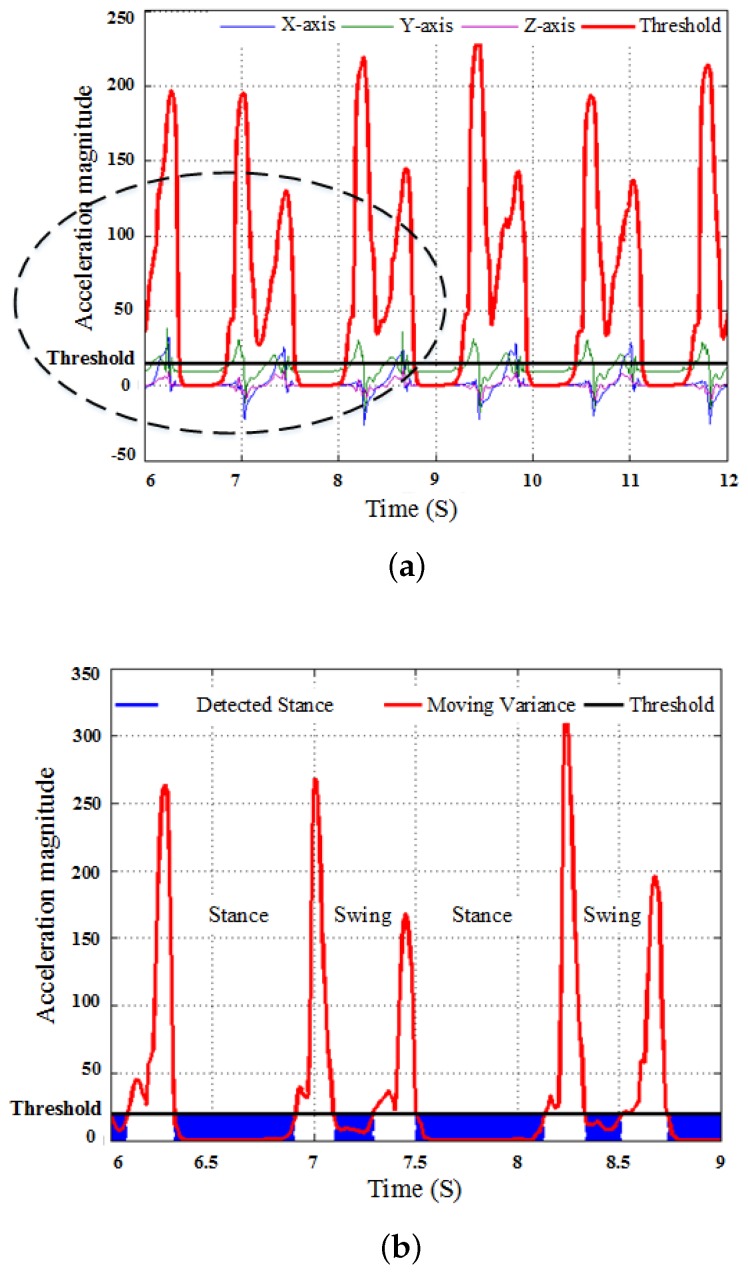
Stance phase detection (**a**) stance phase detection by raw data; (**b**) close-up view.

Sensor drift is an inherent property that results in linear growing integration errors in attitude and position estimation. In particular, position errors grow proportional to the square of the acceleration error. To this end, the widely used Zero Velocity Updating (ZVU) method is adopted in this paper. Though the valid interval of ZVU algorithm is illustrated in literature [31,39,40,41], the effectiveness of the ZVU technique largely relies on the stance phase detection as shown in Figure 3. This paper takes two criteria to determine stance phase in each gait cycle. These key periods are determined by calculating the squared Euclidean norm of acceleration values, as shown in formula (16):

(16) $$V=\sqrt{{\left(a_{x}/∥g∥\right)}^{2}+{\left(a_{y}/∥g∥\right)}^{2}+{\left(a_{z}/∥g∥\right)}^{2}},$$

where $a_{x}$, $a_{y}$ and $a_{z}$ represent the triaxial acceleration measurements of foot sensors:

(17) $$M=\frac{1}{N}\sum_{i=j-N}^{i=j}({\left(S_{i}-{\bar{S}}_{N}\right)}^{2},$$

where ${\bar{S}}_{N}$ is the mean of $S_{i}$ over *N* samples.

Meanwhile, angular rate energy $E_{gyro}$ is adopted as the other criterion (18). The second moment is used to detect stance phase, which is defined in the following formula [39]:

(18) $$E=\frac{1}{\sigma_{\omega }^{2}W}\sum_{i=j}^{j+W-1}{∥\omega_{i}∥}^{2},$$

(19) $$\hat{R}=\left\{\begin{array}{c} 1,V<\lambda_{1}⋂E<\lambda_{2},\\ 0,\;\mathrm{other}\;\mathrm{value}, \end{array}\right.$$

where *W* is the window size selected according to the sensors sampling rate; $\omega_{i}={\left[\omega_{x,i},\omega_{y,i},\omega_{z,i}\right]}^{T}$ is the triaxial angular velocity vector; and $\sigma_{\omega }^{2}$ is the gyroscope noise variance. $\lambda_{1}$ and $\lambda_{2}$ are empirically predefined thresholds. The detection results ($\hat{R}$) are sequences consisting of “zero” and “one”. The algorithm continually finds the interval when ZVU is valid and updates the corresponding $v_{G}\left(t\right)$ as ${\left[0,0,0\right]}^{T}$ based on the two indicators above.

### 3.4. Knee Angle Estimation

**Figure 4:**
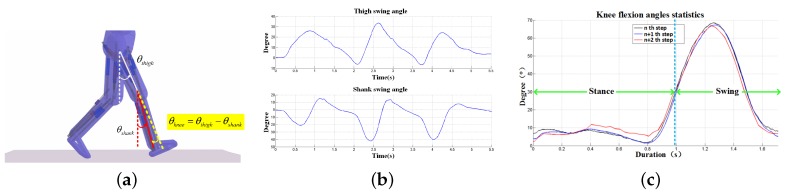
Knee angle estimation results (**a**) calculation principle of knee; (**b**) swing angle of thigh and shank; (**c**) knee flexion flexion statistics.

Knee flexion angle can be calculated by fusing the multi-sensor data. Figure 4a demonstrates the calculation principle and Figure 4b,c show the calculated swing angles and knee flexion based on the gyroscope observations. In addition, the calculation method of knee flexion angle is as follows:

(20) $$\theta_{knee}=\theta_{thigh}-\theta_{shank}+\theta^{ini}=\int \left(\omega_{thigh}-\omega_{shank}\right)dt+\theta_{thigh}^{0}-\theta_{shank}^{0},$$

where $\theta_{thigh}^{0}$ and $\theta_{shank}^{0}$ are initial angles between lower limbs (thigh and shank, respectively) and the gravity direction when the subjects are standing still at ease, which can be calculated by the measurements of the accelerometer as proposed in the previous study [29,30]. $\omega_{thigh}$ and $\omega_{shank}$ are angular velocity values of thigh fixed and shank fixed sensors.

### 3.5. Attitude Estimation and Quaternion Correction in IMUs via Sensor Fusion

**Figure 5:**
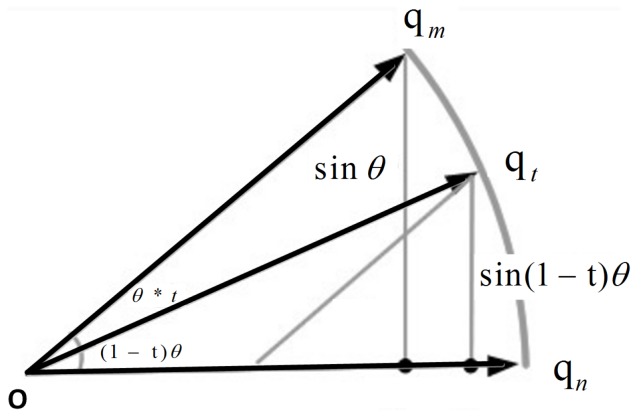
Quaternion interpolation.

IMUs refer to sensor modules consisting of three-dimensional accelerometers, gyroscopes and magnetometers (in some cases magnetometers are not included). According to physical and dynamical theory, acceleration measurements can be integrated once to acquire linear velocity and twice to obtain relative position change based on the previous observations. Likewise, angular velocity observations from gyroscopes can be integrated once to estimate the attitude change between two consecutive measurements. In practice, inertial sensors are prone to be disturbed by system noises, drift and measurement errors. All these factors would cause significant integration errors when the raw sensor data are used for integration. In some cases, when the subject moves slowly or stays static, one can merely adopt accelerometers or inclinometers to directly determine the 3D attitude with acceptable results, which avoids the integration operation. However, it is inevitable that external acceleration applied to the accelerometers could ruin the attitude detection based on the calculation of gravity vector components in these orthogonal planes. In most cases, multiple sensor data fusion is necessary to determine 3D attitude. Since the collected data are discrete, we need to perform interpolation between $q_{m}$ and $q_{n}$ and the interpolation principle is shown in Figure 5.

(21) $$q_{t}=q_{m}+\left(q_{n}-q_{m}\right)\times t,t\in \left[0,1\right].$$

Simple linear interpolation is valid in some cases. However, it can not effectively describe the curve between $q_{m}$ and $q_{n}$. To ensure that the angle θ between $q_{m}$ and $q_{t}$ is linear, i.e., θ(t)=(1−t)θ+tθ, we then choose slerp function $slerp(q_{m},q_{n},t)$ to conduct smooth interpolation of quaternions as follows:

(22) $$slerp\left(q_{m},q_{n},t\right)=\frac{\sin(1-t)\theta }{\sin\theta }q_{m}+\frac{\sin t\theta }{\sin\theta }q_{n}.$$

Normally standardized operation is necessary:

(23) $$q_{t}=\frac{q_{m}+\left(q_{n}-q_{m}\right)\times t}{∥q_{m}+t\left(q_{n}-q_{m}\right)∥}.$$

Moreover, the quaternion number can also avoid the singular point problem of Euler angle representation [27,42], when the pitch angle reaches $\pm 90^{\circ }$. The three-dimensional attitude represented by quaternions are:

(24) $$\phi =\arctan\left(\frac{2\left(q_{2}q_{3}+q_{0}q_{1}\right)}{q_{0}^{2}-q_{1}^{2}-q_{2}^{2}+q_{3}^{2}}\right),$$

(25) $$\theta =\arcsin\left(-2\left(q_{1}q_{3}-q_{0}q_{2}\right)\right),$$

(26) $$\psi =\arctan\left(\frac{2\left(q_{1}q_{2}+q_{0}q_{3}\right)}{q_{0}^{2}+q_{1}^{2}-q_{2}^{2}-q_{3}^{2}}\right).$$

The attitude of the updated sensor can be obtained by solving the differential equation of quaternions. The differential equation of quaternions can be expressed as:

(27) $$\left[\begin{array}{c} {\dot{q}}_{0}\\ \dot{q}{}_{1}\\ {\dot{q}}_{2}\\ {\dot{q}}_{3} \end{array}\right]=\frac{1}{2}\left[\begin{array}{cccc} 0 & -\omega_{x} & -\omega_{y} & -\omega_{z}\\ \omega_{x} & 0 & \omega_{z} & -\omega_{y}\\ \omega_{y} & -\omega_{z} & 0 & \omega_{x}\\ \omega_{z} & \omega_{y} & -\omega_{x} & 0 \end{array}\right]\left[\begin{array}{c} q_{0}\\ q_{1}\\ q_{2}\\ q_{3} \end{array}\right].$$

On the basis of the widely used sensor coordinate transformation, we can get the updated attitude quaternion with inevitable errors. As for a certain vector, its magnitude should be the same though it is expressed in different coordinate frames. The magnitude deviation caused by coordinate transformation can be adopted to adjust the rotation matrix. This paper uses two reference vectors (gravity vector and magnetic vector) to modify the quaternion. In the static state (without linear acceleration), gravity vector ${\left[0,0,1\right]}^{T}$ is converted into ${\left[c_{x},c_{y},c_{z}\right]}^{T}$ after coordinate transformation, while accelerometer observations are ${\left[a_{x},a_{y},a_{z}\right]}^{T}$. Then, ${\left[c_{x},c_{y},c_{z}\right]}^{T}$ and ${\left[a_{x},a_{y},a_{z}\right]}^{T}$ both represent the gravity vector in the sensor frame. Then, we can obtain error matrix $e_{g}^{s}$ by multiplying both vectors:

(28) $$e_{g}^{s}=\left[\begin{array}{c} e_{x}\\ e_{y}\\ e_{z} \end{array}\right]=\left[\begin{array}{c} a_{x}\\ a_{y}\\ a_{z} \end{array}\right]\times \left[\begin{array}{c} c_{x}\\ c_{y}\\ c_{z} \end{array}\right]=\left[\begin{array}{c} a_{y}*c_{z}-a_{z}*c_{y}\\ a_{z}*c_{z}-a_{x}*c_{z}\\ a_{x}*c_{y}-a_{y}*c_{x} \end{array}\right].$$

The error matrix can be used to correct the attitude quaternion. Proportional-Integral (PI) feedback adjustment is introduced hereby,

(29) $$\omega_{t}=\omega_{t-1}+k_{p}*e_{g}^{s}+k_{i}*e_{g}^{s}*\tau ,$$

where $\omega_{t}$ is a three-axis angular velocity component, which can be used to correct quaternions combined with skew symmetric matrix. $k_{p}$ and $k_{i}$ are proportion coefficient and integral coefficient, respectively. Both parameters are ascertained after repeated experiments on different specific groups.

**Figure 6:**
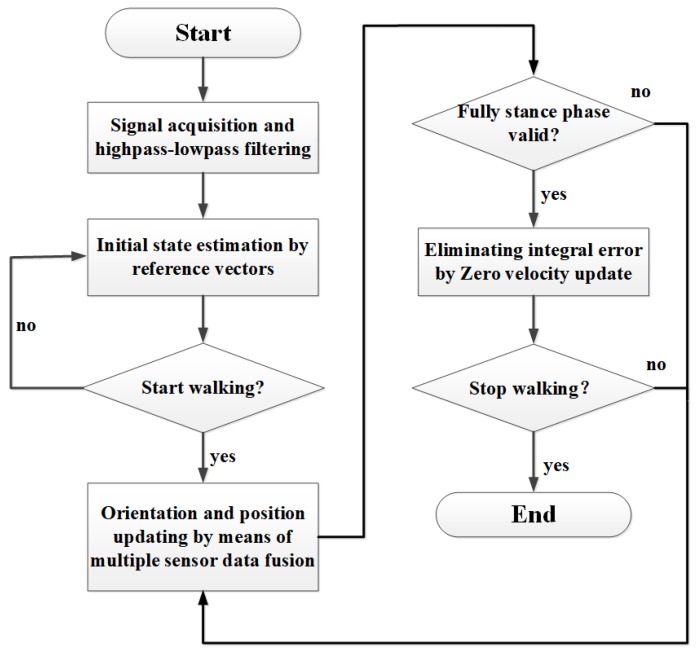
Flowchart of the proposed approach.

Moreover, in practice, the measurement error of MEMS magnetometer is non-negligible, the magnetometer measurement error mainly includes environmental interference and inherent error [12,37]. Researchers have conducted various calibration methods using high precision instruments and equipments [38,43,44], however, these methods are time-consuming and the calibration effect largely relies on the precision of equipment. This work uses a calibration method which allows non-experts to easily implement the calibration procedure. To calibrate the magnetometer, sensor nodes were rotated along "Figure of eight knot" trajectory for several times before they were mounted on human lower limbs. Then the outputs were wirelessly collected and be used to perform the magnetometer correction. The correction of magnetometer should be performed near the body segment where the sensor was mounted in an indoor experimental scene [45]. In conclusion, the flowchart of the proposed gait parameters estimation approach can be summarized in Figure 6.

## 4. Experimental Results

The proposed gait analysis system has been adopted to carry out preliminary clinical trials at the Second Affiliated Hospital of Dalian Medical University. Gait data used in this study consist of walking stride time series from 30 healthy adults (22–45 years old), 20 patients with stroke (46–77 years old), and 20 patients with joint disease (30–58 years old). All subjects were instructed to walk continuously on level ground along an obstacle-free corridor for more than 15 m. Note that we have conducted walking trials including U-turns and stair climbing into the evaluation and the proposed method works well. Straight walking is a simplified case suggested by the clinician, and straight walking is mostly adopted in the observational method in clinical practice.

According to the obtained gait parameters in Table 4, the stride lengths, stride speed and feet clearance are relatively low in both neurological and arthropathy patients, which are also consistent with clinical observation. Similarly, mean values of stance time associated with patients are significantly higher than those of healthy subjects. The results from statistical results presented in the table indicate strong evidence of the capability of the typical gait parameters in characterizing the walk of healthy subjects and patients. In this regard, these gait-related symptoms can be explained by clinicians for diagnostic and treatment purposes because the disease progression can be quantificationally monitored via these observations.

### 4.1. Knee Flexion Monitoring

Table 5 presents the maximum of joint angle during walking. The knee flexion angle is constantly positive; the positive ankle joint angle represents dorsiflexion and a negative value signifies plantarflexion. In the course of stance phase, the knee flexion angle increases; meanwhile, the ankle dorsiflexion turns to plantarflexion; note that the maximum ankle plantarflexion appears in the final stage of stance phase; in the course of swing phase, knee flexion angles reach the maximum while ankle plantarflexion turns to dorsiflexion, followed by the next stance phase.

**Figure 7:**
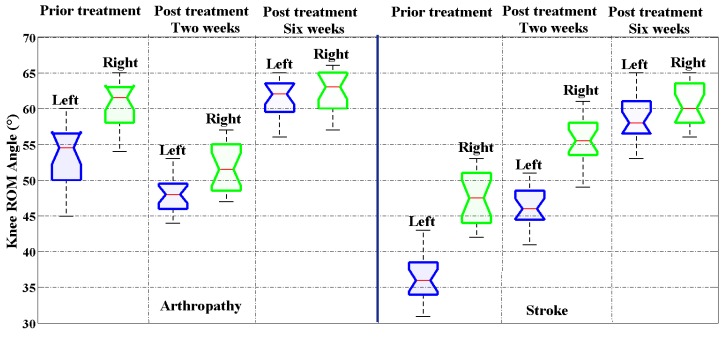
Knee range of motion (ROM) recovery history before and after medical treatments for an arthropathy patient and a stroke patient, respectively.

Knee flexion range of motion (ROM) is often evaluated using a goniometer in rehabilitation clinics or in hospital wards. The more knee ROM regained during the therapeutic process, the better knee recovery would be affirmed and the sooner early discharge could be guaranteed. In this study, we conducted data collection and knee ROM analysis on 30 healthy subjects, 20 neurological patients (mainly stroke patients) and 20 arthropathy patients, respectively. The first data collection is performed before medical treatments, while the remaining data collections occurred at two weeks and six weeks after treatments, respectively. Figure 7 illustrates the knee ROM recovery status of one typical arthropathy patient who received minimally invasive surgery and one typical stroke patient undergoing rehabilitation training, respectively. The results show that both patients recovered significantly in terms of knee ROM after receiving six weeks of medical treatments. By six weeks after minimally invasive surgery, the knee ROM of arthropathy patient almost returns to the normal range and the gait symmetry is much better when the pains were alleviated.

The one-way analysis of variance (ANOVA) results of knee ROM between bilateral lower limbs are shown in Table 6 and Table 7. We can conclude that patients showed large standard deviations in knee ROM, which is a significant feature different from healthy subjects. In this paper, the hypothesis is the symmetrical (balanced) bilateral knee angle ROM, *p*-value <0.05 should be interpreted as the hypothesis is true, and the hypothesis is invalid for subjects with *p*-value >0.05. Results showed that, as for arthropathy patients, no significant knee ROM difference exists on bilateral lower limbs after six weeks’ treatment based on the ANOVA analysis results (*p*-value = 0.0046); however, the stroke patient still has significant knee ROM different on bilateral lower limbs after six weeks of treatment (*p*-value = 0.8637). In fact, many stroke patients still have obvious asymmetry between bilateral knee ROM even after several months, though the symptoms may be relieved to a great extent.

### 4.2. Feet Clearance Monitoring

**Figure 8:**
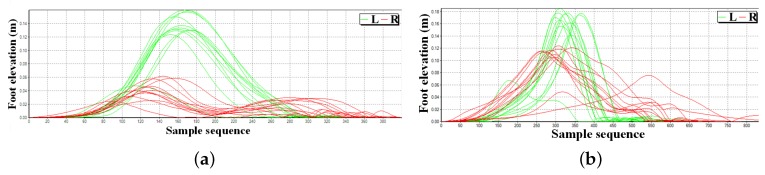
Gait clearance comparison (**a**) a stroke patient; (**b**) an arthropathy patient.

According to Figure 8a, it is observed that there exists foot elevation asymmetry (left foot: ∼15 cm, right foot: ∼5 cm) of a stroke patient with hemiplegia symptoms on the right foot, which is consistent with clinical observation, indicating that the patient loses the ability to keep balance. Figure 8b presents the foot elevation asymmetry of an arthropathy patient, which presents the gait walking disorder from the perspective of feet elevation statistics.

## 5. Discussion and Conclusions

This paper aims to provide ambulatory and robust measurements of human gait, and we adopted body-worn sensors to estimate gait parameters. Digitalized and objective gait information can act as desirable guidance for making and adjusting rehabilitation plans, and the results of the preliminary clinical gait analysis experiments have also verified the accuracy of this method for human limbs motion capturing. With no pressure sensor for the stance phase detection and no optical device for integral error elimination, the pure ZVU-aided gait analysis system using body-worn IMU can achieve a good auxiliary diagnosis performance.

**Figure 9:**
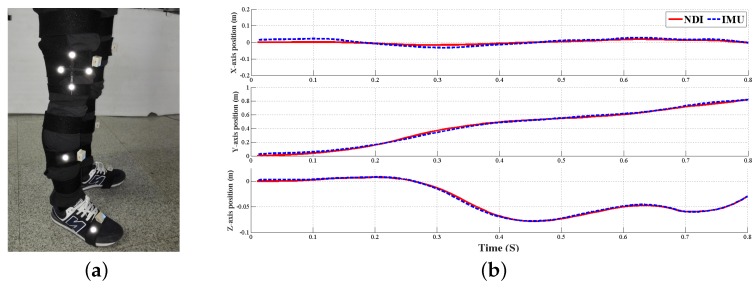
System accuracy validation by an NDI Polaris Spectra System (**a**) sensor placement and reflection points of optical system; (**b**) error statistics of a three-dimensional thigh sensor position estimation for random lower limbs’ movement.

Experimental studies have been presented for an Magnetic Angular Rate and Gravity (MARG) unit with reference measurements obtained via a precision optical measurement system, i.e., the NDI Polaris Spectra System (Northern Digital Inc., Waterloo, ON, Canada). The proposed gait analysis system accuracy was validated and the three-dimensional position estimation error is less than 0.015 m, as shown in Figure 9. A comparison experiment with an optical system has demonstrated the accuracy and feasibility of the proposed principle of error correction, and the results of the preliminary clinical gait analysis experiments have also verified the accuracy of this method for human limbs motion capturing.

The current work provides new insights to better understand the biomechanics of walking due to neurological diseases. In addition, they appear to be valuable tools that can highlight differences in gait dynamics with respect to stroke patients. In this regard, measurement of gait may possibly afford pertinent clinical information on neuromotor conditions, characterization of some neurological disorders, and rehabilitation. A better understanding of gait differences based on etiology of amputation or fall history may provide useful information to help guide prosthetic prescription or rehabilitation interventions.

## Figures and Tables

**Table 1:** Typical spatio-temporal gait parameters.

Gait Parameter	Description
Stride length (m)	Distance between two consecutive footprint of the same foot.
Stride speed (m/s)	Stride length divided by walking cycle.
Stride frequency	Number of steps taken per minute during walking.
Walking cycle (s)	Duration of a single stride, inversely proportional to cadence.
Stance time (s)	Duration of stance phase when feet contact with the ground, starting with initial-contact (IC) and ending with foot-off (FO) of the same foot.
Swing time (s)	Duration of swing phase when feet swing above the ground, starting with FO and ending with IC.
Clearance (m)	Foot elevation in swing phase, which reflects the muscular strength of lower limbs and can be diversified as maximum and minimum foot elevation.
Plantar & dorsiflex (degrees)	The angle between the dorsum of the foot and the back of the leg.
Knee ROM (degrees)	Range of knee flexion during a single stride.

**Table 2:** Comparison of mainstream gait analysis method.

Items	Observation Method	Optical System	Inertial Body Sensor Network (BSN)
Objectivity	subjective	objective	objective
Robustness	poor	sensitive to occlusion	very stable
Repeatability	poor	high	high
Efficiency	medium	low	high
Set-up time	several minutes	half-hour	several minutes
Usability	high	low	high
Visual text	no	partial	fully

**Table 3:** Sensor performance specification.

Unit	Accelerometer	Gyroscope	Magnetometer
Dimensions	3 axes	3 axes	3 axes
Dynamic Range	±50 m/s${}^{2}$	±1200 deg/s	±750 mGauss
Bandwidth (Hz)	30	40	10
Linearity (% of FS)	0.2	0.1	0.2
Bias stability (unit 1σ)	0.02	1	0.1
Alignment Error (deg)	0.1	0.1	0.1

**Table 4:** Gait parameters comparison for healthy subjects and patients. Results are presented as mean (±SD).

Parameter	Healthy	Neurological	Arthropathy
Stride length (m)	1.21±0.13	0.68±0.35	0.73±0.29
Stride speed (m/s)	0.94±0.15	0.71±0.26	0.86±0.21
Stride frequency	92±9	64±22	72 ±17
Walking cycle (s)	1.32±0.08	1.68±0.13	1.48±0.07
Stance time (s)	0.86±0.05	1.14±0.11	0.99±0.08
Swing time (s)	0.46±0.03	0.54±0.06	0.49 ±0.04
Clearance (m)	0.22 ±0.04	0.08 ±0.07	0.14 ±0.06
Knee ROM (degrees)	65±9	39±17	46±22

**Table 5:** Joint angle calculation results of a healthy subject and a typical stroke patient. Results are presented as mean (±SD).

Joint Angle °	Heel Strike	Foot Flat	Heel Off	Swing
Knee joint (Healthy subject)	7.2±3.6	19.9±5.1	39.4±6.1	66.2±5.4
Knee joint (Stroke Patient)	9.8±4.5	14±4.2	22.3±5.8	38.1±7.1
Ankle joint (Healthy subject)	8.7±4.4	15.3±4.3	−19.5±6.8	−8.3±4.2
Ankle joint (Stroke Patient)	9.3±4.5	11.4±5.1	−16.9±5.6	−7.2±3.9

**Table 6:** Analysis of variance (ANOVA) table of bilateral knee range of motion (ROM) for an arthropathy patient (SS: Sum of squares of variance; df: Degree of freedom of variance; MS = SS/df; F: F test statistic).

Item	Source	SS	df	MS	F	*p*-Value
Prior treatment	Columns	285.1	19	15.0053	0.39	0.9787
	Error	778	20	38.9		
	Total	1063.1	39			
Post treatment 2 weeks	Columns	260.275	19	13.6987	1.26	0.3061
	Error	217.5	20	10.875		
	Total	477.775	39			
Post treatment 6 weeks	Columns	227.275	19	11.9618	3.39	0.0046
	Error	70.5	20	3.525		
	Total	297.775	39			

**Table 7:** Analysis of variance (ANOVA) table of bilateral knee range of motion (ROM) for a stroke patient.

Item	Source	SS	df	MS	F	*p*-Value
Prior treatment	Columns	304.28	19	16.0145	0.23	0.9988
	Error	1385.5	20	69.275		
	Total	1689.78	39			
Post treatment 2 weeks	Columns	203.9	19	10.7316	0.21	0.9993
	Error	1006	20	50.3		
	Total	1209.9	39			
Post treatment 6 weeks	Columns	165.6	19	8.7158	0.6	0.8637
	Error	290	20	14.5		
	Total	455.6	39			

## Abbreviations

The following abbreviations are used in this manuscript:

MEMS	Micro-Electro-Mechanical Sensor
BSN	Body Sensor Network
IMU	Inertial Measurement Unit
MARG	Magnetic Angular Rate and Gravity
ZVU	Zero Velocity Updating
